# Challenges of setting up flow cytometry for diagnosis of leukemia and lymphoma at Moi Teaching and Referral Hospital, Eldoret, Kenya

**DOI:** 10.11604/pamj.2015.20.101.5690

**Published:** 2015-02-04

**Authors:** Lotodo Teresa, Kigen Nikol, Patel Kirtika, Injera Wilfred

**Affiliations:** 1 School of Medicine, Moi University, code 30100, Eldoret, Kenya

**Keywords:** Flow Cytometry, leukemia, cluster of differentiation(CD)

## Abstract

The bone marrow (BM) is a complex tissue containing cells of multiple hematopoietic cell lineages in all stages of development. Flow cytometric immunophenotyping evaluates the frequencies of the various leukocyte (sub) populations in BM and blood that then helps in the diagnosis of leukemia's. The aim of this study was to identify challenges of setting up an advanced diagnostic tool like flow cytometry in a resource strained country like Kenya. This is based on a 2 year experience of use of flow cytometry for diagnosis of leukemia, the challenges faced by both the pathologists and the technical team.

## Letter to PAMJ editors

Flow cytometry is a system for sensing cells or particles as they move in a liquid stream through a laser light beam past a sensing area. The relative light scattering and colour-discriminated fluorescence of the microscopic particles is [1]. This diversity of application can only be effectively made in the full knowledge of patient circumstances, however. There are a wide range of applications for flow cytometry in a number of different disciplines. However, in haematology it has become an important tool in the identification of haematological disorders from a wide range of diagnostic samples, such as peripheral blood, bone marrow, CSF, pleural effusion, ascitic fluid and lymph node aspirates. For the analysis of solid tissue a cell suspension must first be made. So far since a year ago, we have made a diagnosis of about two hundred patients despite many challenges that are outlined below [2].

**Clinical background:** lack of clinical background information pertaining the patient, so that appropriate antibodies can be used enabling effective use of resources. For example if clinicians suspect multiple myeloma then appropriate antibodies i.e CD20, CD45, CD138,CD38 and CD56 are used. For burkitts lymphoma then the following antibodies will be used; CD5,CD23, CD20,CD10 and CD45.

**Sampling issues**: peripheral blood and bone marrow aspirates need to be collected in anticoagulant tubes EDTA or heparin zed, but some samples are collected in plain tubes hence rejected. The type of sample needs to be indicated in the collection tube whether peripheral blood or BMA and any other sample, sample type is important in the interpretation especially of blast population. Volume of the sample in the collection tube is paramount in determining sample intergrity since sample volume to anticoagulant ratio is crucial. Peripheral blood less than 2ml in a 4ml EDTA tube, clotted or haemolysed should be rejected. Aspirate taken too early after induction for acute leukemia are often hypocellular, blood dilute and non-informative thus clinicians should be aware of this phenomenon. CSF/ascetic tap and FNA samples have relatively low cell numbers even when a patient has leukemia/lymphoma. Acquisition of 100,000 or more cells by the machine is adequate for interpretation of the sample.

### Technical issues

**Operator training:** flow cytometer is a complex instrument, clearly adequate operator training is essential in terms of producing reliable results. The experience and competency of the cytometrist is critical in analyzing and presenting data for interpretation actually there is lack of experienced cytometrist in the organizations. The choice of optimal gating strategy requires a considerable experience and some knowledge of the diagnosis for example gating on CD34 versus SSC is commonly used in suspected acute leukemia to estimate blast cell population, however a number of acute leukemia are CD34 negative. Knowledge of these entities and appropriate alternative gating strategy is essential and a better approach is CD45 versus SSC in the identification of CD45 dim cells but keeping in mind some leukemias may lose CD45 expression [3].

**Antibody and fluorochrome selection:** it is useful to have a selection of antibodies with different flourochromes to enable multicolour analyses in a variety of panels, but it is unfortunate that some antibodies supplied have same flourochrome making multicolour analyses in a variety of panels impossible e.g CD45 antibody which is virtually crucial is conjugated to PE while CD10,CD20,CD33 and MPO are too conjugated to PE then impossible to analyse B lymphoblasts or myeloblasts [4]. Cytometrist should be aware of situation where two antibodies are directed at adjacent epitopes that can mechanically interfere with each other. Some commercial antibodies e.g CD15 are directed at different epitopes on the antigen and their results are not necessarily comparable when studying monocytes. Spectral overlap is a frequent error in flow signal thus it requires operator experience in instrument compensation setting [5].

**Background fluorescence:** cytometrist should be aware of autoflourence and non-specific antibody binding which are mostly experience as cells age explaining why old samples are rejected or not examined. Non-specific binding can be minimized by using optimal PH buffer, incubation, appropriate ratio of antibody to cells and thorough washing after incubation

**Quality control**: commercially fixed whole blood with established reference ranges should be available to enhance internal quality control together with commercial standards.

**Interpreting results**: perhaps the single most important caveat in cytometry immunophetyping is the failure to correlate with morphological assessment. The cytometrist should be aware of abberant expression of markers, underexpression, overpression and loss of expression of markers in interpretating results.

Flow cytometry is an important tool in diagnosis of hematological disorders especially malignancies but if above mentioned challenges are not well addressed then it may not be beneficial for management of patients.

**Recommendation:** the design of the laboratory request form and the data supplied is therefore of utmost importance and should gather all relevant clinical information that might direct intelligent use of the flow cytometer. The laboratory should participate in external quality assurance scheme for immunophetyping of leukemia and lymphoma. Flow cytometry should not be done in isolation but in conjuction with other available methods like morphology, cytogenetics and other molecular tests, their results should be integrated into a comprehensive final pathology report and cases of discrepancies should be discussed in a wide forum.
